# The Association between Frailty and Inflammatory Biomarkers in the Elderly

**DOI:** 10.1093/geroni/igaf122.2886

**Published:** 2025-12-31

**Authors:** Xiaotian Shi, Qing Ma, Wen Tang, Shan Wang, Huayu Yang, Deqiang Zhang, Yuanyuan Li, Ying Sun

**Affiliations:** Beijing Friendship Hospital, Capital Medical University, Beijing, Beijing, China (People’s Republic); Beijing Friendship Hospital, Capital Medical University, Beijing, Beijing, China (People’s Republic); Beijing Friendship Hospital, Capital Medical University, Beijing, Beijing, China (People’s Republic); Beijing Friendship Hospital, Capital Medical University, Beijing, Beijing, China (People’s Republic); Beijing Friendship Hospital, Capital Medical University, Beijing, Beijing, China (People’s Republic); Beijing Friendship Hospital, Capital Medical University, Beijing, Beijing, China (People’s Republic); Beijing Friendship Hospital, Capital Medical University, Beijing, Beijing, China (People’s Republic); Beijing Friendship Hospital, Capital Medical University, Beijing, Beijing, China (People’s Republic)

## Abstract

**Objectives:**

This study aims to characterize associations between frailty and systemic inflammation using blood-derived indices and cytokines, and identify causal mechanisms through proteogenomic integration.

**Methods:**

We analyzed neutrophil-to-lymphocyte ratio (NLR), derived NLR (dNLR), monocyte-to-lymphocyte ratio (MLR), and hemoglobin-to-red cell distribution width ratio (HRR) from routine blood tests. Serum interleukin-6 (IL-6), IL-1β, IL-18, and tumor necrosis factor-α (TNF-α) were quantified. Mendelian randomization (MR) integrated plasma proteome-wide genome-wide association study (GWAS) data (N = 54,219) with UK Biobank frailty genetics (n = 175,226) for causal inference. Functional annotation of differentially expressed proteins employed Gene Ontology (GO) and Kyoto Encyclopedia of Genes and Genomes (KEGG) pathway analyses.

**Results:**

Adjusted analyses identified NLR (OR = 1.57, 95%CI=1.07–2.30), dNLR (OR = 1.59, 1.11–2.27), and HRR (OR = 0.13, 0.03–0.44) as independent frailty predictors. Elevated IL-6 (OR = 1.14, 1.01–1.30) and TNF-α (OR = 1.21, 1.07–1.36) correlated with frailty. MR revealed 26 plasma proteins causally linked to frailty, including five inflammation-associated proteins. Pathway analysis demonstrated JAK-STAT signaling enrichment, connecting chronic inflammation to frailty pathogenesis.

**Conclusions:**

NLR, dNLR and HRR serve as accessible frailty biomarkers, while IL-6 and TNF-α are cytokine drivers of frailty. The MR-validated plasma proteome implicates JAK-STAT axis activation as a key mechanism driving inflammation-associated frailty, providing therapeutic targets for this geriatric syndrome.

